# *Callicarpa stoloniformis* (Lamiaceae), a new species from Southeast China based on morphological characters and phylogenetic evidence

**DOI:** 10.1002/ece3.9913

**Published:** 2023-03-23

**Authors:** Zhonghui Ma, Xiangxiu Su, Huimin Cai, Zhiwei Su, Bin Chen

**Affiliations:** ^1^ College of Agriculture, State Key Laboratory for Conservation and Utilization of Subtropical Agro‐bioresources, National Demonstration Center for Experimental Plant Science Education Guangxi University Nanning China; ^2^ Feng Yang Seedling Plantation Pingnan County Fujian Province China; ^3^ Institute of Marine Drugs Guangxi University of Chinese Medicine Nanning China; ^4^ Eastern China Conservation Center for Wild Endangered Plant Resources, Shanghai Chenshan Botanical Garden Shanghai China

**Keywords:** *Callicarpa*, Lamiaceae, morphology, phylogenetic analysis

## Abstract

*Callicarpa stoloniformis* sp. nov. (Lamiaceae) is described as a new species from Fujian Province of China on the basis of both morphological and molecular data. The new species is morphologically most close to *C. hainanensis*. However, it can be distinguished from the latter by its unique procumbent life form, adventitious roots at nodes, papery leaves, cup‐shaped or campanulate calyx, truncate or shallow fissure calyx lobes, and smaller fruits. In addition, the new species is also similar with *C*. *basitruncata*, a species only known from the original description and the photograph of holotype, but it can differ from the latter by its procumbent shrub, purple terete branchlets with apparent linear lenticels, adventitious roots at nodes, and papery larger leaves with prominently cordate leaf base. Original photographs, illustration, distribution map, and a comparative morphological table, as well as an identification key of the related taxa are provided.

## Graphical abstract

*Callicarpa stoloniformis* sp. nov. (Lamiaceae) is described as a new species from Fujian Province of China on the basis of both morphological and molecular data. Unique procumbent life form and adventitious roots at nodes make it distinctly different from other species in *Callicarpa*.

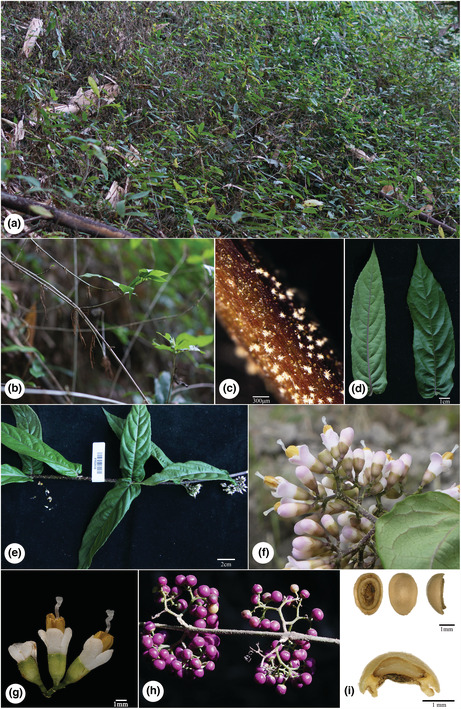

## 1 INTRODUCTION

*Callicarpa* L. (Linnaeus, 1753: 111, Lamiaceae) with her nickname “beauty berry” is mainly distributed in tropical, subtropical, and temperate Asia, America, Australia, and some Pacific Islands (Bramley, 2009, 2013; Harley et al., 2004). There are about 140 species of *Callicarpa* recorded around the world, with 33 species recognized in the New World, particularly in the Caribbean Islands (24 species in Cuba). Obviously, the genus is more species‐rich in the Old World, which was inferred as the origin center by the newest molecular phylogeny (Liu et al., 2022). Specifically, ca. 48 species were recognized in Temperate Asia, particularly in China (45 species and 12 varieties, Bramley, 2009, 2013; Ma, 2013). In addition, there were ca. 51 species recorded in Malesia, seven species in Australia (Munir, 1982), three species in the Pacific, and one species in Madagascar (Moldenke, 1950). *Callicarpa* has a long history as a member of Verbenaceae after being assigned by Robert Brown (1810). Based on numerous morphological and molecular phylogenetic studies, *Callicarpa* was transferred to Lamiaceae, along with several other genera, such as *Premna* L. (Linnaeus, 1771: 154) and *Tectona* L. (Linnaeus, 1753: 151) (Harley et al., 2004). Recently, evidences from palynology (Ma et al., 2016) and molecular phylogeny (Li et al., 2016; Zhao et al., 2021) further corroborated the sister relationship between *Callicarpa* and the Australian endemic subfamily Prostantheroideae, and then, a new subfamily Callicarpoideae was established (Li et al., 2016; Zhao et al., 2021).

Attempts to make a more scientific infrageneric classification for *Callicarpa* have been based on various morphological characters. According to calyx characters, Briquet (1895) divided the genus into two different groups: section *Tubulosae*, defined by tubular calyx with deeply 4‐fid rim, and long, generally foliaceous lobes; section *Cyathimorphae*, characterized by campanulate or cyathiform calyx with a subtruncate and entire or only shortly 4‐(or 5‐) toothed rim. Subsequently, Chang (1951) found some examples that fell between Briquet's groups during his investigation of Chinese species. Then, he divided *Callicarpa* into two sections based on stamen features: section *Eucallicarpa* and section *Verticirima*. The former was characterized by small, ovate, longitudinally dehiscent anthers, with filaments twice or more than twice as long as the corolla. The latter was defined by its larger oblong anthers and filaments that are not longer than the corolla. Afterward, when compiling Flora Reipublicae Popularis Sinicae, Fang (1982) divided *Callicarpa* into two subgenera based on hair types: Subgen. *Peiantha*, composed of a single taxon, *C*. *peichieniana* (1982: 78), which was characterized by strongly curved stem hair; while other species with various hair types were assigned to Subgen. *Callicarpa*. In recent years, evidences from morphology, palynology, and molecular phylogeny have proved that there was a sister relationship between *Callicarpa* and subfamily Prostantheroideae endemic in Australia and both formed a basal clade in Lamiaceae (Cai et al., 2021; Li et al., 2016; Ma, 2013; Ma et al., 2015; Ma & Su, 2015; Ma & Zhang, 2012; Zhao et al., 2021). Recently, more comprehensive phylogenic studies on the framework of Lamiaceae suggested establishing a new monotypic subfamily Callicarpoideae (Zhao et al., 2021). Liu et al. (2022) combined the analysis of phylogeny, biogeography, and statistics, and revealed that the fruit color of *Callicarpa* was strongly associated with geographical distribution. Besides, they also suggested all fruit colors of the genus were involved in dispersal events, and inferred violet fruit promoted diversification in *Callicarpa* and drove the evolution and diversity of different fruit colors between regions. In Asia, a number of *Callicarpa* are valued traditional medicinal plants and several of them, such as *C. formosana* (Trimen et al. 1882: 358), *C. macrophylla* (Vahl & Forssk, 1794: 13), *C. kwangtungensis* (Chun, 1934: 302), and *C. nudiflora* (Hooker et al. 1841: 206) have been intensively studied and included in Chinese Pharmacopoeia (2020 edition). Furthermore, drug products of them are widely used in clinical (eg. Luo Hua Zi Zhu Pian, Kang Gong Yan Pian, Fu Yan Ning Jiao Nang, Wu et al., 2018). Chinese Pharmacopoeia Commission, (2020).

As part of our ongoing taxonomic revision of *Callicarpa* in China, some interesting specimens were collected from Neikengkou, Nanjing County (Fujian Province, China). After examination of the floras or monographs from China and the adjacent Asian regions (Bramley, 2009, 2013; Chen & Gilbert, 1994; Fang, 1982; Leeratiwong et al., 2009; Ma, 2013), as well as analysis of herbarium specimens, our discovered plants cannot be placed in none of the current known species of *Callicarpa*. Based on morphological and molecular phylogenetic data, we decided to propose the newly collected plants as a new species.

## 2 MATERIALS AND METHODS

### 2.1 Morphology

Field surveys of the putative new species were carried out in Neikengkou, Nanjing County, Zhangzhou City, Fujian Province, during the period of June–December 2021. Syndromes of the habitat, life form, hairs, leaves, cyme, and fruit were surveyed with naked eyes or a hand magnifier and photographs were taken in the field. Detailed characters of glandular, hair, floral anatomy, ovary, and seed were observed with stereomicroscope (NIKON SMZ25) in the laboratory. Examination of specimens was also made for morphological comparison with the related species (herbaria visited: GAUA, IBSC, PE, CSH, IBK, NY, G, E, A, P, TI, and K; acronyms according to Thiers, 2022). The size of leaves, cymes, flowers, and fruits was measured with a vernier caliper in the laboratory. All voucher photos of the putative new species were deposited in the “Chinese Field Herbarium” (https://www.cfh.ac.cn/album/ShowSpAlbum.aspx?spid=94377).

### 2.2 Molecular phylogeny

A total of 14 species representing the main sections, subsections, and series of Chinese *Callicarpa* were selected for molecular phylogenetic analyses with two species from Prostantheroideae: *Dasymalla teckiana* (Conn et al. 2011: 6) and *Dicrastylis parvifolia* (Mueller, 1861: 160) as the outgroups. Total DNA was extracted from fresh leaves dried with silica gel using a modified CTAB method (Doyle & Doyle, 1987). Two nuclear [internal transcribed spacer (ITS) and external transcribed spacer (ETS)] and five chloroplast (*matK*, *rpl32*‐*trnL*, *trnH*‐*psbA*, *psbJ*‐*petA*, and *trnS*‐*trnG* intergenic spacer) regions were chosen for phylogenetic analyses. PCR amplification, sequencing, sequence editing, and sequence assembly were performed according to Katoh and Standley (2013) and Kumar et al. (2016). The primer pairs used for PCR were shown in Table 1. Detailed information on the DNA regions and GenBank accession numbers of the species investigated in this study were shown in Table 2.

**TABLE 1:** Primers used for phylogenetic analyses.

Marker	Primer	Sequence (5′ to 3′)	References
ITS	17SE	ACGAATTCATGGTCCGGTGAAGTGTTCG	Sun et al. (1994)
	26SE	TAGAATTCCCCGGTTCGCTCGCCGTTAC	
ETS	18S‐IGS	GAGACAAGCATATGACTACTGGCAGGATCAACCAG	Baldwin and Markos (1998)
	ETS‐B	ATAGAGCGCGTGAGTGGTG	Beardsley and Olmstead (2002)
*matK*	323f	ATTNTCAAATCNTAKCAGAGGGG	Andersson (2006)
	1189r	CGGCTTACTAATRGGATGCCC	
*rpl32*‐*trnL*	trnL(UAG)	CTGCTTCCTAAGAGCAGCGT	Shaw et al. (2007)
	rpL32‐F	CAGTTCCAAAAAAACGTACTTC	
*trnH*‐*psbA*	psbA F	GTTATGCATGAACGTAATGCTC	Sang et al. (1997)
	trnH2	CGCGCATGGTGGATTCACAATCC	Tate and Simpson (2003)
*psbJ*‐*petA*	psbJ	ATAGGTACTGTARCYGGTATT	Shaw et al. (2007)
	petA	AACARTTYGARAAGGTTCAATT	
*trnS*‐*trnG*	trnS (GCU)	AGATAGGGATTCGAACCCTCGGT	Shaw et al. (2005)
	5′trnG2S	TTTTACCACTAAACTATACCCGC	

**TABLE 2:** Information of voucher and GenBank accession numbers for sequence data.

Taxon	Coll. No.	Collector(s)	Location	ETS	ITS	*matK*	*psbJ*‐*petA*	*rpl32*‐*trnL*	*trnG(UCC)‐trnS (GCU)*	*trnH*‐*psbA*
*C. japonica 14294*	K‐LCD_1934‐12904	Bramley et al.	Thailand	ON931539	ON820162	OP032150	OP032157	OP081587	OP032168	OP032163
*C. brevipes MZH44*	ZHM0130	Zhonghui Ma	Dalin mountain, Guangdong, China	ON931491	ON820123	OP032115	OM460815	OP081586	OP032173	OP032167
*C. hainanensis MZH22*	ZHM079	Zhonghui Ma	South China Botanical Garden, China	OM307542	OM333849	OM630173	OM460810	OM501619	OM403845	OM473350
*C. longipes MZH9*	ZHM085	Zhonghui Ma	Nankun mountain, Guangdong, China	OM307549	OM333856	OM630178	OM460805	OM501625	OM403851	OM473340
*C. integerrima* var. *chinensis MZH19*	8887	X.X. Huang	Jiulian mountain, Jiangxi, China	OM307530	OM333838	OM630183	OM460802	OM501631	OM403857	OM473347
*C. rubella MZH6*	ZHM089	Zhonghui Ma	Wutong mountain, Guangdong, China	OM307557	OM333864	ON964474	OP032159	OM530212	OM403906	OM473339
*C. stoloniformis 02816*	02816	Xiangxiu Su	Nanjing County, Fujian, China	OP032174	OP030615	OP032175	OP032176	OP032177	OP032178	OP032179
*C. poilanei 23,191*	2596	Suddee et al.	Ubon Ratchathani, Nam Yuen dist, Thailand	OM307603	OP135611	OM530154	OM439786	OM501603	OM403827	OM439784
*C. arborea MZH42*	ZHM099	Zhonghui Ma	Xishuangbanna, Yunnan, China	ON931488	ON820119	OP032112	OP032161	OP081584	OP032171	OM489764
*C. candicans MZH20*	LB0162	Bo Li	Hainan, China	OM307537	OM333844	OM630169	OM460814	OM501614	OM403840	OM473348
*C. giraldii MZH12*	LB0233	Bo Li	Yunnan, China	ON931499	ON820130	OP032121	OP032160	OP081585	OP032170	OP032165
*C. nudiflora MZH69*	ZHM0155	Zhonghui Ma	Bawanglin, Hainan, China	OM307553	OM333860	OM630181	OM460801	OM501629	OM403855	OM489771
*C. longifolia MZH43*	ZHM0117	Zhonghui Ma	Xishuangbanna, Yunnan, China	ON931514	OM333855	OP032131	OP032162	OP081583	OP032172	OP032166
*C. pentandra 25,527*	SAN147213	Bramley et al.	Bombalai Hill, Malaysia	ON931526	ON820150	OP032141	OP032158	OP081588	OP032169	OP032164
*Dasymalla teckiana*			Australian National Botanic Gardens (ANBG), Australia	#	#	NC_058334	NC_058334	NC_058334	NC_058334	NC_058334
*Dicrastylis parvifolia*			Australian National Botanic Gardens (ANBG), Australia	#	GQ381162	NC_058335	NC_058335	NC_058335	NC_058335	NC_058335

Maximum likelihood (ML) and Bayesian inference (BI) were conducted for the phylogenetic analyses by IQ‐TREE (Nguyen et al., 2015) and MrBayes (Ronquist et al., 2012) in Phylosuite v1.2.2 (Zhang et al., 2020) and MrBayes on XSEDE3.2.7a as implemented in CIPRES (http://www.phylo.org/) (Miller et al., 2010) respectively. The bootstrap (BS) percentage for each branch was estimated by running 1000 bootstrap replicates. For BI analysis, ModelFinder (Kalyaanamoorthy et al., 2017) was used for the selection of the most appropriate evolutionary model (nucleotide substitution model) (Edge‐linked) using BIC criterion. The best partition models with BIC criterion were as follows: for ETS and ITS, HKY + F + G4; for *matK*, *psbJ‐petA*, *rpl32*‐*trnL* and *trnG*‐*trnS* intergenic spacer, GTR + F + G4; for *trnH*‐*psbA* intergenic spacer, F81 + F. The run with 1,000,000 generations was conducted. Four Markov chains with two runs were implemented and sampled every 100 generations, and the first 25% of all trees were regarded as “burn‐in”. The majority consensus of the remaining trees was generated to show posterior probability (PP) support for clades. Stationarity was determined in Tracer v1.7.1 (Rambaut et al., 2018) and was considered to be attained when ESS > 200 or when the average standard deviation of the split frequencies was <0.01.

## 3 RESULTS

### 3.1 Morphology

**FIGURE 1:**
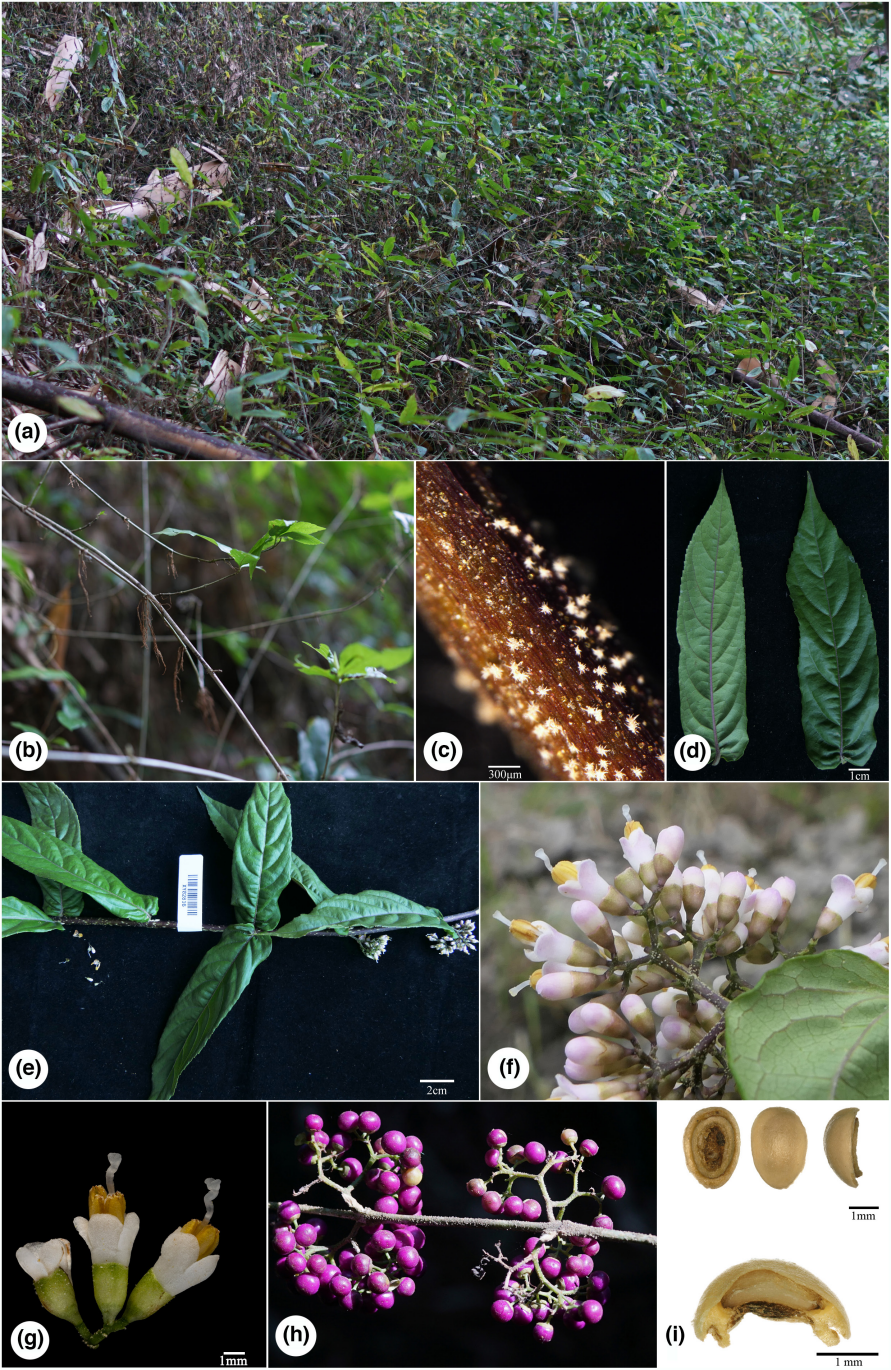
*Callicarpa stoloniformis*. (a) Habit; (b) branchlet with adventitious roots at nodes; (c) branchlet with stellate tomentose; (d) adaxially and abaxially of blade; (e) stem with leaves and cymes; (f, g) inflorescence; (h) fruit; (i) pyrenes.

**FIGURE 2:**
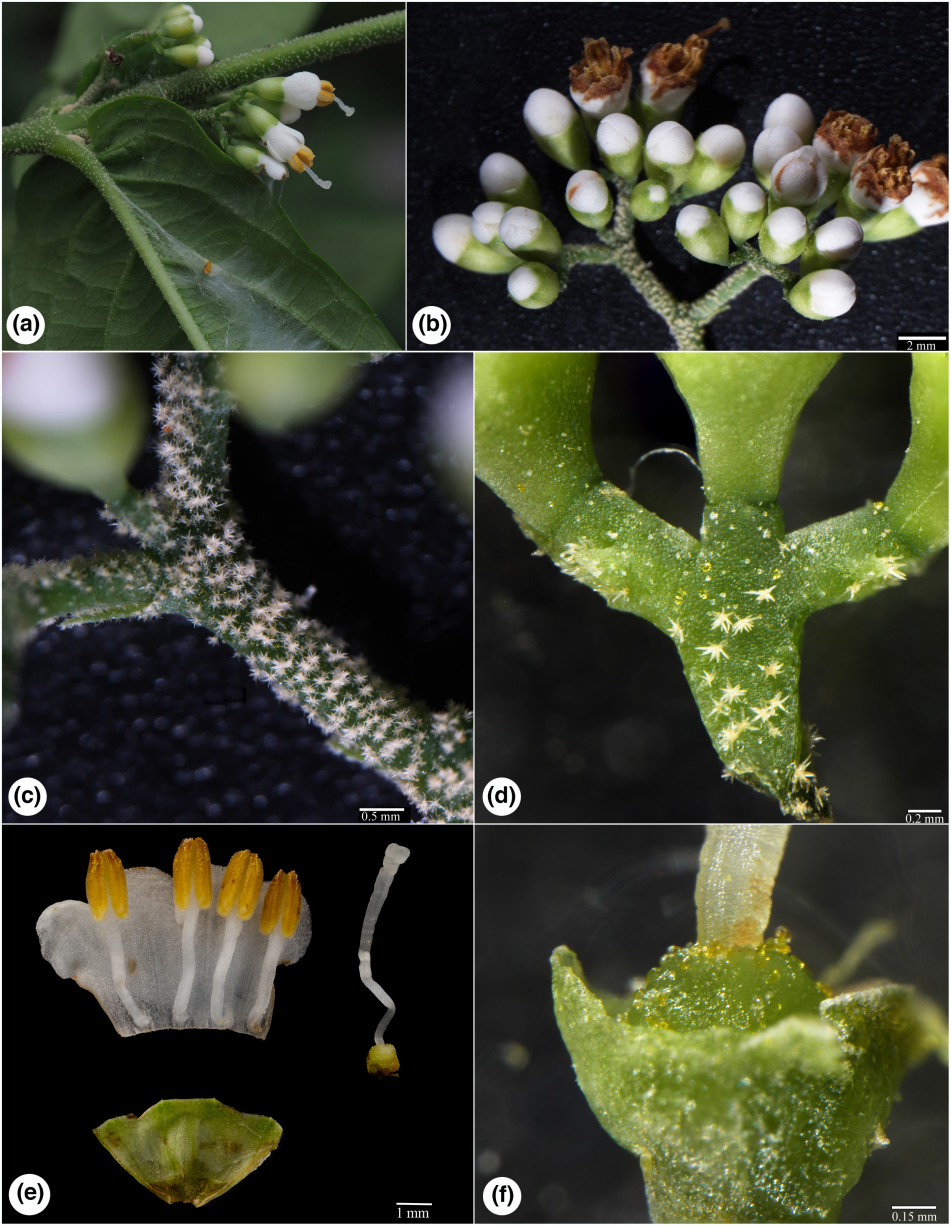
Inflorescence of *Callicarpa stoloniformis*. (a, b) Influorescence; (c, d) peduncle and pedicel with stellate tomentose and yellow glandular; (e) corolla (split open); (f) ovary.

**FIGURE 3:**
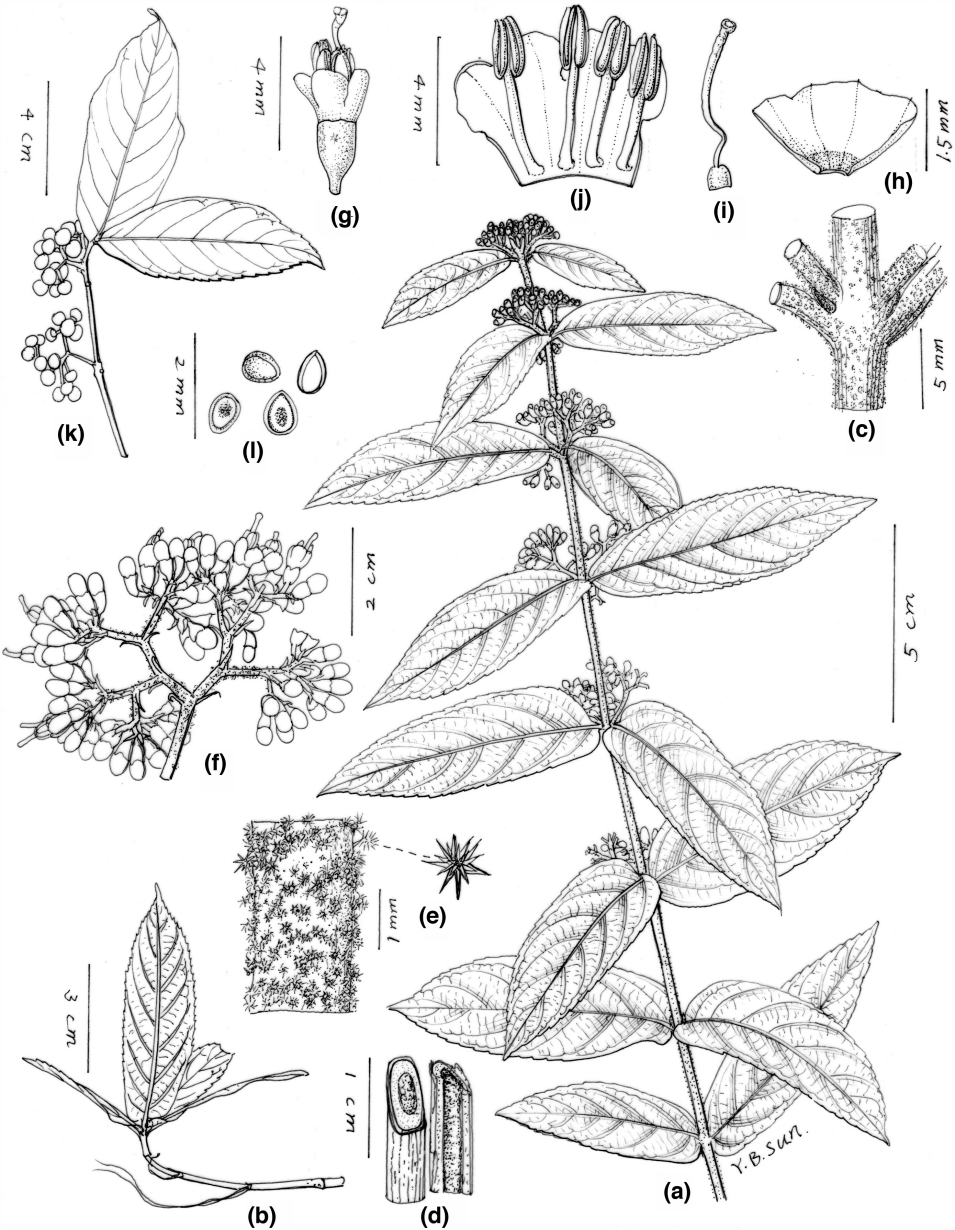
Illustration of *Callicarpa stoloniformis*. (a) Plant with flowers; (b) branchlet with adventitious roots at nodes; (c) detail of stem; (d) cross‐ and longitudinal section of stem showing the pith; (e) stellate tomentose on the branchlet; (f) cyme; (g) flower; (h) calyx; (i) ovary with style; (j) corolla (split open) with stamens; (k) infructescence; (l) pyrenes with seed inside.

The putative new species is similar to *Callicarpa hainanensis* Ma & Zhang (2012: 573) and *C. basitruncata* Merrill ex Moldenke (1951: 406), and their detailed morphological comparison from life form, stem, leaves, cymes, calyx, corolla, and fruit were list in Table 3. The new species is most similar to *C. hainanensis*, and both share lots of common characteristics in branchlets, indumentum, leaf base, filament length, and the way of anther opening. Even so, it can be distinguished from *C. hainanensis* by its unique life form, adventitious roots at nodes, the morphology of calyx, calyx lobes, and the size of fruits (Table 3, Figures 1, 2, 3). In addition, the putative new species also resembles *C. basitruncata*, and they all have similar leaf shapes, cymes, and calyx, but it can differ from the latter by its procumbent life form, terete branchlets with adventitious roots at nodes, apparent linear lenticels, and leaf morphology (Table 3, Figures 1, 2, 3). In terms of leaf blade base, the putative new species is slightly similar to *C*. *rubella* and *C*. *longipes*, which all have cordate leaf bases. Obviously, it has filaments slightly shorter or as long as corolla, with larger oblong anthers, while the latter two species all have filaments twice or more than twice as long as the corolla, with small, ovate anthers.

**TABLE 3:** Morphological comparison of *Callicarpa stoloniformis* with two related species.

Character	*C. stoloniformis*	*C. hainanensis*	*C. basitruncata*
Life form	Procumbent shrub	Erect shrub	Erect shrub
Stem	Terete branchlets, purple, with stellate tomentose (when young) and linear lenticels, with adventitious roots at nodes	Terete branchlets with stellate tomentose and linear lenticels, no adventitious roots at nodes	Obtusely tetragonal branchlets, light‐colored, lenticels unconspicuous, no adventitious roots at nodes
Leaves	Leaves petiole ca. 2–5 mm long, wrinkled, papery, oblong lanceolate, 11.5–14.5 × 3.5–4.5 cm, apex acuminate, base cordate	Leaves subsessile, subcoriaceous, obovate‐lanceolate, 15–20 × 3–5 cm, apex acuminate, base subcordate or auriculate‐semiamplexicaul	Petiole 1–2 mm, membranous, oblong, or slightly oblanceolate, 6–9 × 1–2 cm, apex acute or subacuminate, base truncate
Cymes	3–4 branched	3–4 branched	Usually 2–3 branched
Calyx	Cup‐shaped or campanulate, glabrous, with densely yellow glandular, do not dehisce as fruits mature, calyx lobes truncate or rare shallow fissure	Long cup‐shaped or subtubular, tube sparsely stellate pubescent, yellow glandular, dehisced as fruits mature, calyx lobes sharply triangular, ca. 2 mm	Cup‐shaped, very sparsely stellate puberulent to glabrescent, very sparsely glandular, calyx lobes obscurely 4‐toothed to almost truncate
Corolla	White to pink, ca. 4‐mm‐long	White, ca. 5‐mm‐long	White, ca. 2‐mm‐long
Fruit	ca. 1.5–2 mm in diameter	ca. 4 mm in diameter	ca. 2 mm in diameter

### 3.2 Molecular phylogeny

**FIGURE 4:**
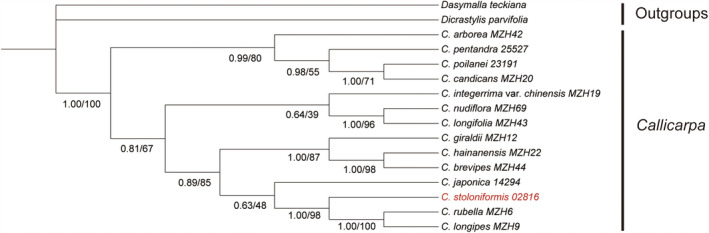
Phylogenetic relationships among *Callicarpa stoloniformis* and representative species of *Callicarpa* based on the combined DNA data (two nuclear and five chloroplast regions). The numbers under the nodes are Bayesian posterior probabilities and maximum likelihood bootstrap percentages, respectively.

The cpDNA dataset comprised an aligned matrix of 3958 base pairs (bp) with 814 bp for *matk*, 1046 bp for *psbJ*‐*petA* intergenic spacer, 791 bp for *rpl32*‐*trnL* intergenic spacer, 742 bp for *trnG*‐*trnS* intergenic spacer, 565 bp for *trnH*‐*psbA* intergenic spacer, and the nrDNA dataset comprised an aligned matrix of 1165 base pairs, with 461 bp for ETS and 704 bp for ITS. The topologies of *Callicarpa* phylogeny generated on the base of combined nrDNA (two loci) and cpDNA (five regions) separately from the ML and BI analyses are not obviously conflict with each other. Here, we present the analysis results based on the combined nrDNA + cpDNA dataset for a better‐solved relationship among the related species investigated. The phylogenetic analysis indicates that the genus *Callicarpa* is monophyletic (posterior probability, PP = 1.00; bootstrap percentage, BP = 100%) with two outgroups from Prostantheroideae. The putative new species forms a clade with *C*. *rubella* Lindl. (Lindley, 1825: 883), *C*. *longipes* Dunn (1908: 363), and *C*. *japonica* Thunb. (Thunberg, 1784: 153), and they form a sister clade with the remaining species of *Callicarpa* (Figure 4).

### 3.3 Taxonomic treatment

***Callicarpa stoloniformis*** X.X. Su, Z.H. Ma & B. Chen, *sp*. *nov*. (Figures 1, 2, 3).

Type: CHINA. Fujian Province: Zhangzhou City, Nanjing County, growing in a mountain valley, elev. ca. 200 m, E117.3643, N24.4886, 14 Dec. 2021, *Xiangxiu Su GBJ09119* (holotype CSH!).

**Diagnosis:** *Callicarpa stoloniformis* is similar to *C. hainanensis* and *C. basitruncata* in stellate indumentum, cordate leaf blade base, filament slightly shorter or as long as corolla and anther opening by an apical pore, but significantly differs from the latter two species by its unique procumbent life form and adventitious roots at nodes (Table 3).

**Description:** Procumbent shrubs, ca. 1 to 2 m tall. Branchlets are slender, purple, terete, with linear lenticels, medullose, yellow glandular, stellate hairs (especially densely on the young branches and nodes) when young, becoming grayish brown and glabrous later with adventitious roots at nodes. Leaves decussate‐opposite, wrinkled, papery; oblong lanceolate, approximately 11.5–14.5 × 3.5–4.5 cm; apex acuminate, base cordate; margin serrate along upper‐middle part; veins abaxially prominent, purple; leaves blade subglabrous, adaxially stellate tomentose on mid‐veins, sparsely yellow glandular, abaxially occasionally stellate tomentose near veins, densely yellow glandular, secondary veins 6–9 pairs, sparsely hairy on lateral veins; petiole short, ca. 2–5 mm long, with densely stellate hairs. Cymes ca. 1.2–1.5 cm across, 3–4 branched; peduncle purple, with densely stellate tomentose, longer than petioles, ca. 5–7 mm long, yellow glandular, bracts linear‐lanceolate; pedicel subglabrous, ca. 1–2 mm long. Calyx cup‐shaped or campanulate, glabrous, with densely yellow glandular, calyx lobes truncate or rare shallow fissure. Corolla white to pink, ca. 4 mm long, glabrous, with yellow glandular; Staments 4, filaments slightly shorter or as long as corolla, anthers oblong, ca. 2.5 mm, opening by an apical pore. Ovary glabrous with yellow glandular. Fruits globose, purple, ca. 1.5–2 mm in diameter, and glabrous (Figures 1, 2, 3).

**Etymology:** The specific epithet is derived from the stoloniform stem.

**Vernacular name:** Simplified Chinese: 匍茎紫珠; Chinese pinyin: Pú Jīng Zǐ Zhū.

**Phenology:** Flowering time August–September; fruiting time September–December.

**FIGURE 5:**
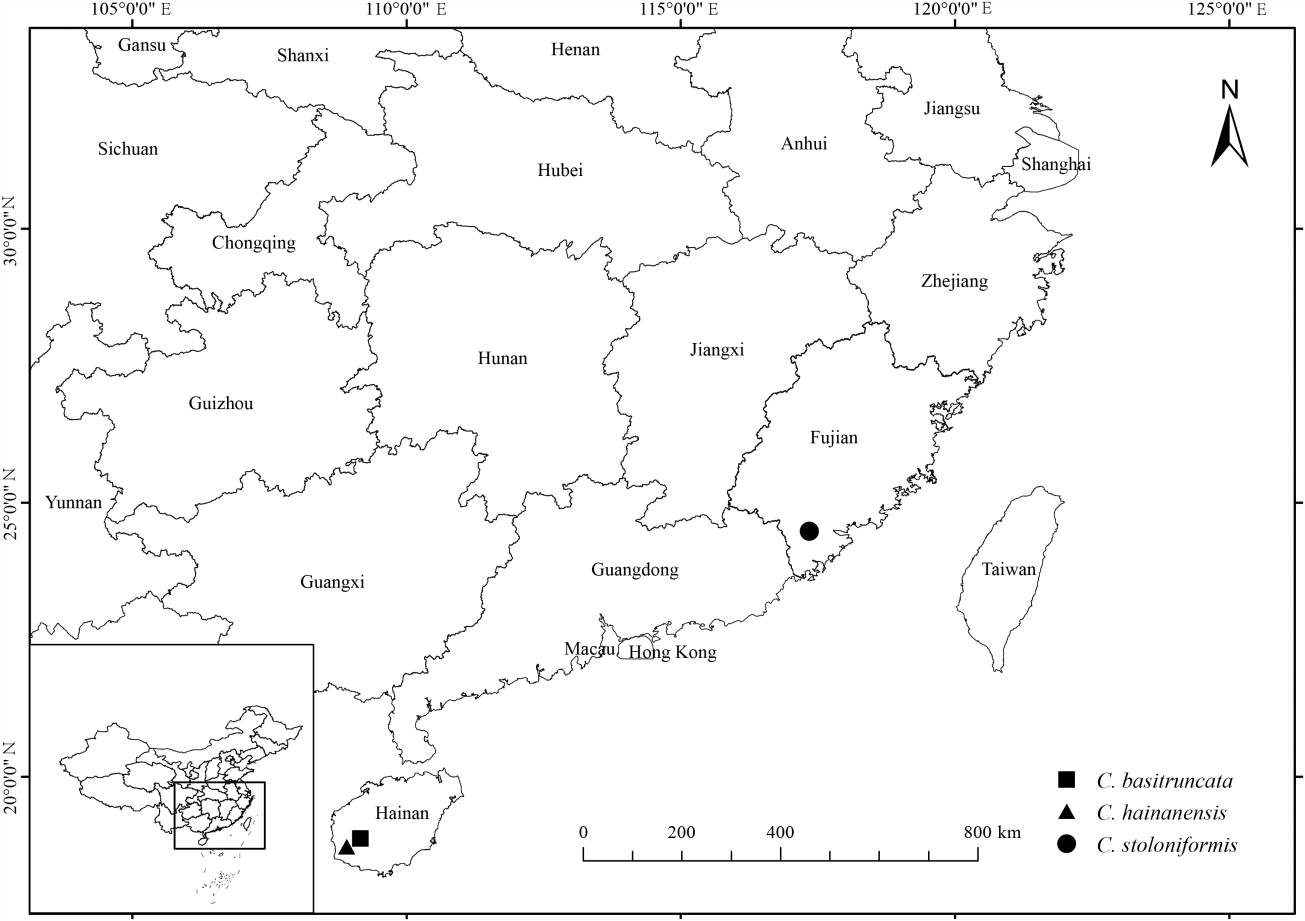
Distribution map of *Callicarpa stoloniformis*.

**Distribution and ecology**: *Callicarpa stoloniformis* is currently known only from *locus classicus* (Neikengkou in Nanjing County, Zhangzhou City, Fujian Province, China) growing in a mountain valley at altitude of 195 m (Figure 5). The new species grows along with *Pinus massoniana* (Don, 1803: 17), *Phyllostachys edulis* (Houzeau de Lehaie, 1906: 39), *Morella rubra* (de Loureiro, 1790: 548), *Alfaropsis roxburghiana* (Iljinskaja, 1993: 82), *Saurauia tristyla* (de Candolle, 1822: 423), *Ficus variolosa* (Hooker, 1842: 492), *Syzygium hancei* (Merrill & Perry, 1938: 242), *Adinandra millettii* (Trimen et al. 1878: 9), *Ternstroemia gymnanthera* (Beddome, 1871: 91), *Alpinia japonica* (1867: 140), *Tetrastigma hemsleyanum* (Diels & Gilg, 1900: 463), *Pellionia radicans* (de Candolle, 1869: 167), *Lophatherum gracile* (Duperrey, 1829: 50), and *Setaria palmifolia* (Stapf, 1914: 186).

#### 3.3.1 Identification key to *Callicarpa stoloniformis* and related taxa

1. Filaments twice or more than twice as long as the corolla, with small, ovate anthers……………………………………………………………………………....2.

1a. Filaments not longer than the corolla, with larger oblong anthers……..………….4.

2. Leaf blade base cuneate, narrowing above the middle………………....… ***C. giraldii***.

2a. Leaf blade base cordate or auriculate‐semiamplexicaul, widest above the middle, obovate oblong or oblanceolate……………………………………………..…..…..3.

3. Calyx teeth acute, 1–2 mm; Petiole 5–10 mm…………………….……… ***C. longipes***.

3a. Calyx teeth obtusely triangular, 0.5 mm; Petiole very short to subsessile………………………………………………………………….. ***C. rubella***.

4. Procumbent branchlets with adventitious roots at nodes………..…. ***C. stoloniformis***.

4a. Upright branchlets without adventitious roots at nodes……………………………5.

5. Branchlets, leaf blade abaxially, peduncle glabrous…………………..… ***C. japonica***.

5a. Branchlets, Leaf blade abaxially veins, peduncle stellate pubescent………………6.

6. Long cup‐shaped or subtubular calyx which dehisces as fruits mature, with sharply triangular calyx lobes………………………………………………… ***C. hainanensis***.

6a. Cup‐shaped calyx which does not dehisces as fruits mature, with subtruncate to minutely 4‐dentate calyx lobes……………………………………..……………….7.

7. Leaf blade base truncate to shallowly cordate………..……..……… ***C. basitruncata***.

7a. Leaf blade base obtuse, cuneate, or shallowly cordate……………….…. ***C. brevipes*.**

#### 3.3.2 Additional specimens examined

CHINA. Shanghai: Shanghai Chenshan Botanical Garden, Collected from cultivated living plant, 8 Oct. 2022, *Bin Chen CB04591* (paratypes GAUA!, CSH!).

#### 3.3.3 Specimens of related species examined

*Callicarpa hainanensis*. CHINA. Hainan: Ledong County, Jianfengling Mountain, alt. 650 m 26 May 2010, *Z. H. Ma 079* (holotype, IBSC!); at the edge of a secondary forest, alt. 500–700 m, 1 June 2010, *Z. H. Ma 090* (IBSC!); on a mountain slope, alt. 520–650 m, 15 September 2010, *Z. H. Ma 113* (IBSC!).

*Callicarpa brevipes*. CHINA. Guangdong: Boluo County, Xiaojinsha, alt. 740 m, 15 Oct. 1992, *Y. Q. Wang 173* (IBSC!); Zhuhai, Dangan, 8 Sept. 1990, *B*. *H*. *Chen 678* (IBSC!); Dalin mountain, 12 Aug. 2009, *ZHM0130* (GAUA!); South China Botanical Garden, 12 May 2012, *ZHM082* (GAUA!); Conghua County, Sanjiao Mountain, 29 May 1932, *W. T. Tsang 20606* (PE!); Heping County, alt. 600 m, 13 Oct. 1984, *G. C. Zhang 630* (IBSC!); Jiangxi: Jiulian mountain, 18 Sept. 2009, *ZHM057* (GAUA!); Hainan: Jianfengling, 16 June 2011, *L*. *X. Zhou 5476* (IBSC!); Jianfengling, Tianchi, 15 Apr. 1982, *Q. Huang 820153* (IBSC!); Fujian: Shunchang County, 7 July 1956, *M. S. Li & Z. Y. Li 5144* (PE!); Guangxi: Longsheng County, 23 Sept. 1956, *S. L. Xu* & *H. F. Qin 700608* (IBK!); Rongxian, Tiantangshan, 26 June 1956, *S. Q. Chen 9581* (IBK!); Wuzhou, 9 June 1935, *S. G. Li 81075* (IBSC!); Bobai County, alt. 820 m, 26 Sept. 1959, *S*. *Q*. *Zhong A63177* (IBSC!); HongKong: *J. G. Champion 12* (K!); *G. Bentham 442* (K!).

*Callicarpa basitruncata*. CHINA. Hainan: 1 Aug. 1935, *J. L. Gressitt 1168* (Type, A!, NY!, G!, E!).

*Callicarpa japonica*. CHINA. Tsinghan: 1 Jan. 1901, *R. Zimmermann 210* (Holotype photo P!); Jiangxi: Yichun, alt. 1400 m, 14 Aug. 1963, *Junsan Yue 3380* (IBSC!); Jiujiang County, alt. 280 m, 28 Sept. 1992, *C. M. Tan 92100‐A* (PE!); Guangchang, 17 Oct. 1962, *Junsan Yue 2544* (PE!); Wuning County, alt. 300 m, 18 June 1963, *S. S. Lai 02688* (PE!); Lushan, alt. 400 m, 27 Oct. 1995, *C. M. Tan 95918* (PE!); Anhui: Huangshan, 12 July 1975, *K. J. Guan 75141* (PE!); Shanxi: Jingcheng, 26 July 1959, *S. Y. Bao 424* (PE!); Zhejiang: 6 June 1958, *Hangzhou 414* (PE!); JAPAN. Minami‐yama, 7 Nov. 2007, *T. Miyazaki 0711004* (TI!); 30 Oct. 2007, *T. Miyazaki 0710534* (PE!).

## 4 DISCUSSION

### 4.1 Morphology

The new species is morphologically similar to *Callicarpa hainanensis* Ma & Zhang by sharing a series of similar features. Specifically, both have terete branchlets with stellate tomentum when young (becoming glabrous when old), and obvious linear lenticels. They have cordate leaf blade bases, slender cymes, with filaments slightly shorter than or as long as corolla, and oblong anthers, opening by an apical pore. However, *C. stoloniformis* can be easily distinguished from *C. hainanensis* due to its procumbent shrub (vs. erect shrub), which make it distinctive from the whole genus. Furthermore, *C. stoloniformis* has adventitious roots at nodes (vs. no adventitious roots at nodes), papery leaves (vs. subcoriaceous), cup‐shaped or campanulate calyx (vs. long cup‐shaped or subtubular), and truncate or shallow fissure calyx lobes (vs. sharply triangular, dehisced as fruits mature) and smaller fruits (1.5–2 mm vs. 4 mm in diameter; Table 3). In addition, the new species is also likely to be confused with *C. basitruncata* Merrill ex Moldenke, a species only known from the original description and a photograph of the holotype. Both species have oblong lanceolate leaf blades, abbreviated cymes, glabrous calyx, truncate or rare shallow fissure calyx lobes. However, it can be distinguished from *C. basitruncata* by its procumbent shrub (vs. erect shrub), terete branchlets (vs. obtusely tetragonal), with adventitious roots at nodes (vs. no adventitious roots at nodes), apparent linear lenticels on stem (vs. lenticels inconspicuous), and papery larger leaves (vs. membranous) with prominently cordate leaf base (vs. truncate) (Table 3). *C. stoloniformis* has a cordate leaf base, which to some extent makes it slightly similar to *C*. *rubella* and *C*. *longipes*, but its shorter filaments (as long as corolla) and larger oblong anthers differ it from the latter two species which have filaments twice or more than twice as long as the corolla, with small, ovate anthers.

### 4.2 Molecular phylogeny

The combined analysis indicated that *Callicarpa* is monophyletic (PP = 1.00, BP = 100%, Figure 4) with respect to the groups considered. Two main clades are formed and our phylogenetic result seems not being consistent with Chang's (1951) traditional classification system based on the length of filament, and morphology of anther, which is widely recognized (Bramley, 2009, 2013; Fang, 1982). The putative new species forms a well‐supported sister clade with the clade composed by members of section *Eucallicarpa*: *C*. *rubella* and *C*. *longipes* (PP = 1.00, BP = 98%), and then, these three species form a weakly supported sister clade with *C*. *japonica* (section *Verticirima*) (PP = 0.63, BP = 48%, Figure 4). Although it forms a sister clade with *C*. *rubella* and *C*. *longipes* and shares few common characteristics in morphology, such as cordate leaf base, it can be obviously distinguished from the latter two species by its unique procumbent life form, adventitious roots at nodes, and shorter filaments or as long as corolla (typical characters of section *Verticirima*). Moreover, it also can be easily identified from *C*. *japonica* by its procumbent life form, adventitious roots at nodes, stellate hairs on the young branchlets and inflorescence, and cordate leaf base. It is strange that *C. hainanensis* which we think the most morphologically similar species with *C*. *stoloniformis* (mentioned above), firstly forms a clade with *C*. *brevipes* (Benth.) Hance (1886: 233) and *C*. *giraldii* Hesse ex Rehd. (Rehder, 1914: 629), subsequently form a moderately supported sister clade with the clade including *C. stoloniformis*, *C*. *japonica*, *C*. *rubella*, and *C*. *longipes* (Figure 4).

## AUTHOR CONTRIBUTIONS

**Zhonghui Ma:** Conceptualization (equal); funding acquisition (equal); writing – original draft (lead); writing – review and editing (equal). **Xiangxiu Su:** Conceptualization (equal); investigation (lead). **Huiming Cai:** Investigation (equal); methodology (equal). **Zhiwei Su:** Conceptualization (equal); funding acquisition (equal); writing – review and editing (equal). **Bin Chen:** Conceptualization (equal); funding acquisition (equal); writing – review and editing (equal).

## CONFLICT OF INTEREST STATEMENT

The authors declare no conflicts of interest.

## Data Availability

The sequences of this study have been deposited in The National Center for Biotechnology Information (NCBI) database. GenBank accession numbers of the sequencing data can be found in Table 2.
